# Experimental infection in calves with a specific subtype of verocytotoxin-producing *Escherichia coli *O157:H7 of bovine origin

**DOI:** 10.1186/1751-0147-51-43

**Published:** 2009-10-31

**Authors:** Malin E Jonsson, Erik Eriksson, Sofia Boqvist, Anne Margrete Urdahl, Anna Aspán

**Affiliations:** 1 National Veterinary Institute, Department for Health Surveillance, Oslo, Norway; 2 National Veterinary Institute, Uppsala, Sweden; 3 Dept of Biomedical Sciences and Veterinary Public Health, Swedish University of Agricultural Sciences, Uppsala, Sweden

## Abstract

**Background:**

In Sweden, a particular subtype of verocytotoxin-producing *Escherichia coli *(VTEC) O157:H7, originally defined as being of phage type 4, and carrying two *vtx*_2 _genes, has been found to cause the majority of reported human infections during the past 15 years, including both sporadic cases and outbreaks. One plausible explanation for this could be that this particular subtype is better adapted to colonise cattle, and thereby may be excreted in greater concentrations and for longer periods than other VTEC O157:H7 subtypes.

**Methods:**

In an experimental study, 4 calves were inoculated with 10^9 ^colony forming units (cfu) of strain CCUG 53931, representative of the subtype VTEC O157:H7 (PT4;*vtx*_2_;*vtx*_2c_). Two un-inoculated calves were co-housed with the inoculated calves. Initially, the VTEC O157:H7 strain had been isolated from a dairy herd with naturally occurring infection and the farm had previously also been linked to human infection with the same strain. Faecal samples were collected over up to a 2-month period and analysed for VTEC O157 by immuno-magnetic separation (IMS), and IMS positive samples were further analysed by direct plating to elucidate the shedding pattern. Samples were also collected from the pharynx.

**Results:**

All inoculated calves proved culture-positive in faeces within 24 hours after inoculation and the un-inoculated calves similarly on days 1 and 3 post-inoculation. One calf was persistently culture-positive for 43 days; in the remainder, the VTEC O157:H7 count in faeces decreased over the first 2 weeks. All pharyngeal samples were culture-negative for VTEC O157:H7.

**Conclusion:**

This study contributes with information concerning the dynamics of a specific subtype of VTEC O157:H7 colonisation in dairy calves. This subtype, VTEC O157:H7 (PT4;*vtx*_2;_*vtx*_2c_), is frequently isolated from Swedish cattle and has also been found to cause the majority of reported human infections in Sweden during the past 15 years. In most calves, inoculated with a representative strain of this specific subtype, the numbers of shed bacteria declined over the first two weeks. One calf could possibly be classified as a high-shedder, excreting high levels of the bacterium for a prolonged period.

## Background

Cattle are regarded as the main reservoir of verocytotoxin-producing *Escherichia coli *(VTEC) O157:H7 [1-3], and play a significant role as a source of human VTEC infections [4-7]. The number of reported domestic human cases of VTEC O157:H7 infection in Sweden between 2000 and 2008 ranged from 28 to 134 annually, the incidence being highest in the southwestern part of the country (personal communication, Sofie Ivarsson, Swedish Institute of Infectious Disease Control (SMI)). Among the human cases of VTEC O157:H7 reported to the SMI, a particular subtype of VTEC O157:H7 has predominated for at least 15 years [8,9]. This particular subtype, that originally was defined as being of phage type (PT) 4, with a particular RFLP pattern of the vt-phage, and carrying the *vtx*_2 _and *vtx*_2c _genes [8] hereafter designated VTEC O157:H7 (PT4;*vtx*_2_;*vtx*_2c_).

More than 2/3 of the VTEC O157:H7 isolates from domestic cases during 2001- 2007 belonged to VTEC O157:H7 (PT4;*vtx*_2_;*vtx*_2c_) (personal communication, Sven Löfdahl, SMI). It has also been the causative agent in two large food-borne outbreaks in Sweden; one outbreak in 2003 due to cold fermented sausages including 30 reported cases [10] and one outbreak in 2005 where 135 cases, including 11 HUS patients, were culture positive for VTEC O157:H7 after consumption of fresh lettuce [11].

Studies in Sweden have shown that the prevalence of VTEC O157:H7 in faecal samples collected from cattle at slaughter is 3.4% [12] and that, similar to the human incidence, the prevalence in cattle is highest in southwestern Sweden [13,14]. Furthermore, it has been established that high cattle density is a risk factor for human VTEC O157:H7 infection [14]. Generally, calves have a higher prevalence of VTEC O157:H7 colonisation, excrete the agent at greater concentrations than adult cattle [15,16] and the shedding declines with increasing age [12,17].

Several experimental studies have found that the faecal shedding of VTEC O157:H7 varies in magnitude and duration among individual animals [15,18-21]. It has been suggested that persistence of infection within a cattle herd may require the presence of one or several animals that shed large numbers of the bacterium over an extended period of time, and that identification and isolation of these animals is necessary to prevent and control VTEC O157:H7 at farm level [22]. Such animals would also pose a high risk of direct and foodborne transmission of the infection from animals to man. A suggested definition for high-shedding is a count of at least 10^3 ^colony-forming units (cfu) per gram of faeces, persisting for at least 2 weeks [22]. It has earlier been reported findings of VTEC O157:H7 from the pharynx in cattle [23]. However, in another study [24] VTEC O157:H7 was not isolated from the oral cavity. In view of those contrasting findings it would be interesting to investigate the occurrence of the bacterium in the oral cavity in cattle further.

Currently, there is an ongoing discussion in Sweden about how to prevent the spread of VTEC O157:H7, for example by a control programme that will lower risk of transmission by restricting animal trade from "high-risk farms" (VTEC O157:H7 detected in environmental samples) to " low-risk farms" (VTEC O157:H7 not detected in environmental samples). Therefore, knowledge about the magnitude and duration of shedding of the specific subtype VTEC O157:H7 (PT4;*vtx*_2_;*vtx*_2c_) and whether it is similar to that reported for other VTEC O157 in other studies, is of importance. The aim of this study was to investigate the magnitude and duration of faecal excretion in calves experimentally infected with VTEC O157:H7(PT4;*vtx_2_*;*vtx_2c_*) and of transmission of the bacterium between calves.

## Methods

### Experimental animals

Seven preweaned castrated bull calves of the Swedish red-and-white breed, aged between 6 and 9 weeks, were obtained from a conventional dairy farm belonging to the Swedish University of Agricultural Sciences, Uppsala, Sweden. All calves were deemed healthy by clinical examination and haematological analyses (haemoglobin level, white blood cell count and differential leukocyte count; data not shown). Furthermore, faecal samples were tested, with negative results, for coronavirus and rotavirus (21 and 13 days pre inoculation), *Cryptosporidium *sp. (13 days pre inoculation), *Salmonella *spp. (5 days pre inoculation) and three times for VTEC O157:H7 (13, 7 and 1 days pre inoculation). To assess health status during the experiment, general condition and rectal temperature were monitored daily, and body weight once a week. The animals were fed on concentrate appropriate to their age and breed, and had free access to hay as well as water of drinking quality through water nipples. Ethical approval was obtained from the Ethical Reviews Committee (Uppsala, Sweden).

### Experimental design and housing facilities

The study was performed during a 2-month period and throughout the experiment the calves were housed in a bio-containment level 3 facility at the National Veterinary Institute, Uppsala, Sweden. They were randomly assigned to three groups, housed under loose conditions in three separate stalls, and were given identification numbers I-VII; calves I, II, III in stall A, calves IV, V, VI in stall B and calf VII in stall C as a negative control. Transmission of bacteria via water, feed, manure, ventilation, tools or instruments between the stalls was impossible, and personnel changed boots and protective clothing between stalls.

Before the experiment started the calves were acclimatised to the isolation facility over a 10-day period. Each stall had a floor area of 12 m^2^, individual floor drain and separate ventilation system. The bedding material was sawdust. The stalls were cleaned twice a day, in the morning by removing all sawdust and faeces from the floor, and hosing the floor and walls with warm water; in the afternoon only faeces and wet spots were removed from the floor.

Of the calves, 2 from each stall were randomly assigned (calves II and III in stall A and calves IV and V in stall B) to be inoculated with 10^9 ^cfu of VTEC O157:H7. On day 42 pi, calf V was re-inoculated and calf VI was inoculated for the first time, both with 10^9 ^cfu of the same strain. Calves were euthanized on day 14 pi (calves III and IV), day 48 pi (calf VII), day 49 pi (calves I and II) and on day 55 pi (calves V and VI).

### Bacterial strain

The VTEC O157:H7 isolate used in this experiment was obtained in 1999 from faeces of a 10-month-old calf on a conventional Swedish dairy farm. The farm was implicated in a human VTEC O157:H7 infection in 1997. Genotyping with Pulsed-Field Gel Electrophoresis (PFGE), as described by Gautom [25] showed that the strain, present over time in faecal samples from calves on the farm, belonged to the genetic cluster of VTEC O157:H7 (PT4;*vtx*_2;_*vtx*_2c_) that has predominated in human infections in Sweden, as described above. The strain has been deposited in the Culture Collection of Gothenburg University (Göteborg, Sweden) and been assigned accession number CCUG 53931.

### Inoculation of animals

To prepare the inocula, the strain CCUG 53931 was cultivated at 37°C for 18 hours in nutrient broth (Oxoid, Basingstoke, England) supplemented with 10% bovine serum. The concentration of VTEC O157:H7 in the enrichment broth was tested to be 1 × 10^9 ^cfu/ml by serial dilution and plating. Four inocula were prepared by transferring 1.0 ml of the broth to 25 ml 0.1% peptone water (Oxoid) and immediately administered to the calves by peroral gastric intubation.

### Sampling

Faecal samples were collected every second day the first 2 weeks after inoculation, followed by sampling twice a week for the remainder of the experiment. Also, samples from the pharynx were collected once a week. Faecal samples were collected by digital rectal retrieval and pharyngeal samples by using a sterile cotton-tip swab (Transystem, Amies with charcoal, Copan Italia, Brescia, Italy). All samples were collected in the afternoon and stored in refrigerated state. The analyses were started within 24 h of sampling.

### Isolation and typing of VTEC O157

VTEC O157:H7 was isolated from faeces by immunomagnetic separation (IMS) on 10 g faeces, as described elsewhere [13]. Isolation of VTEC O157:H7 from swab samples was effected by pre-enriching each swab in 5 ml pre-warmed (37°C) BPW for 6-8 h at 37°C. Isolates were confirmed by PCR for the presence of verocytotoxin 2, intimin, enterohaemorrhagic *E. coli*-hemolysin genes, as described by Paton and Paton [26] and the flagellar antigen H7 gene according to Gannon et al [27]. PFGE was carried out on altogether 28 isolates. Samples for PFGE analyses from the calves in stall A were taken on days 3, 9, 19, 33 and 47 pi. The first two samplings included all calves, and the three latter, calves II and III. From stall B, samples subjected to PFGE were sampled on days 3 and 9 pi (all calves) and on days 2, 4, 6 and 8 pi re-infection (calves V and VI).

### Quantitative analysis

The quantitative analysis was initiated within 24 h of sampling on samples culture-positive on IMS. Enumeration of *E. coli *O157-positive faecal samples was performed by serially diluting 10 g faeces in 0.1% peptone water. Duplicate aliquots of 0.1 ml from different dilutions, selected according to the results of the previous count, were plated onto CHROMagar O157 plates (CHROMagar, Paris, France) supplemented with potassium tellurite (1.25 mg/ml), hereafter called ChromO157, and incubated at 37°C for 21-24 h. Numbers of presumptive *E. coli *O157 colonies were recorded and five such colonies from each sample were confirmed by latex agglutination with O157 antiserum. The detection limit by this method was estimated to 1 × 10^2 ^cfu/g faeces (data not shown). Results from samples deemed culture-positive by IMS, but negative by the quantitative analysis, were considered positive by IMS, while samples culture negative by IMS were deemed negative.

## Results

### Experimental animals

All calves remained healthy throughout the study period and maintained a normal appetite, normal rectal temperature, were alert and responsive, and had a normal growth curve (data not shown).

### Isolation of VTEC O157:H7 in faeces

**Figure 1:**
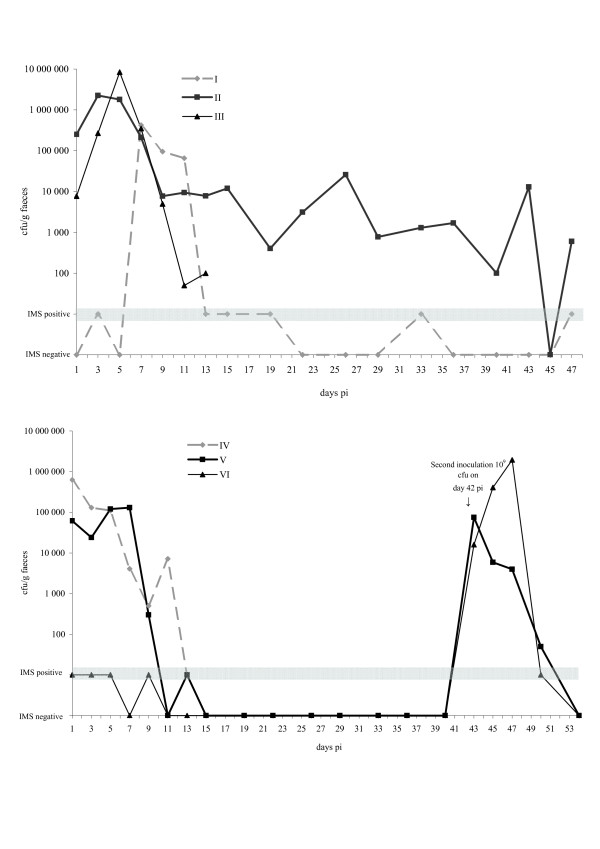
**Time course of faecal excretion of VTEC O157:H7, strain CCUG 53931, in experimentally inoculated calves**. Excretion is shown for calves I, II and III in stall A (upper panel) and for calves IV, V and VI in stall B (lower panel). Calf I was left un-inoculated. Calf VI was inoculated for the first time on day 42. On that same day, calf V was re-inoculated. Samples negative on counting but culture-positive on IMS are denoted as IMS positive (grey bar), while samples culture-negative on IMS are denoted as IMS negative. The calves were euthanised after the last shown result of sampling.

All 4 inoculated calves were culture positive for VTEC O157:H7 strain CCUG 53931 in faeces within 30 h pi, rates ranging from 7.7 × 10^3 ^to 6.3 × 10^5 ^cfu/g faeces (Fig. 1). Maximum shedding ranged from 1.3 × 10^5 ^to 8.4 × 10^6 ^cfu/g for the different calves, taking place between day 1 and day 7 pi. The numbers of VTEC O157:H7 shed in faeces decreased over the first 15 days pi. Calf II was culture-positive in faeces for 43 days (16 consecutive samplings). The 2 un-inoculated calves, I and VI, turned culture-positive on days 3 and 1 pi, respectively.

On day 42 pi, when the 2 remaining calves in stall B (V and VI) had been culture-negative for 8 and 10 consecutive samplings respectively, they were inoculated with 10^9 ^cfu of the same VTEC O157:H7 strain. Both calves were culture-positive the next day (43 pi). The shedding rate decreased quickly and on day 54 pi (day 12 after second inoculation) both calves were negative. Calf VII, the control calf kept in stall C, was negative on all sampling occasions.

### Isolation of VTEC O157:H7 in pharyngeal samples

Altogether 30 swab samples from pharynx were analysed for VTEC O157:H7 and all were negative.

### PFGE typing of isolates

**Figure 2:**
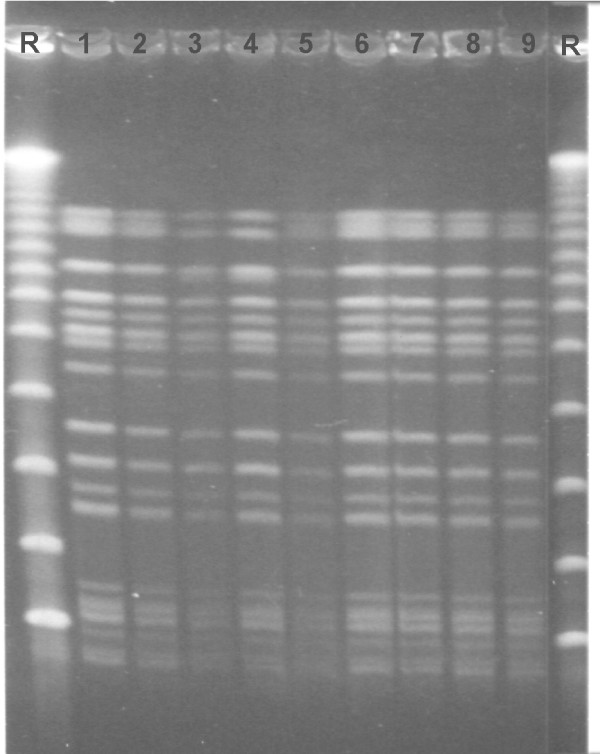
**Macrorestriction profiles following *Xba*I cleavage and Pulsed-field gel electrophoresis**. Macrorestriction profiles following *Xba*I cleavage and Pulsed-field gel electrophoresis of calf isolates of VTEC O157:H7. Two distinct pulsotypes are seen. R represents size standards lambda ladder; New England Biolabs. Pulsotype 1: lanes 1-2 and 5-9; Pulsotype 2: lanes 3-4.

Altogether 28 isolates from the experimentally infected calves were subjected to PFGE. Of these 28 isolates, 26 had pulsotypes identical to the inoculum strain (CCUG 53931). For two isolates, from faecal samples of calf V and calf VI on day 5 after the second inoculation, a slightly different pulsotype was observed (Fig. 2).

## Discussion

In this experimental study, the shedding pattern of a specific strain of VTEC O157:H7 in cattle was investigated. The strain was chosen as a representative of the VTEC O157:H7 (PT4;*vtx*_2_;*vtx*_2c_) subtype, and was isolated from cattle on a farm previously linked to direct transmission of infection to man. This particular subtype of VTEC O157:H7 is prevalent among cattle in Sweden [12] and predominates among the human cases reported to the SMI [8]. There have been two larger human outbreaks of VTEC O157:H7 in Sweden, both caused by this subtype of VTEC O157:H7 [10,11].

In the present study the shedding patterns following experimental infection in different calves were very similar, and for most of the calves shedding ceased within two weeks. Previous experimental and field studies have shown large variations in the magnitude and duration of VTEC O157:H7-shedding in beef and dairy cattle. However, in most studies the same pattern is observed; after an intial period of shedding in high levels there is a decline towards lower levels whitin 2-3 weeks, except for a small subset of animals where shedding may persist at high levels for longer periods [15,20,28,29]. One of the experimentally infected calves shed the bacterium for 43 days. For practical reasons, a high-shedding animal has been defined as excreting enough bacteria to be detected by direct plating, i.e. between 10^3 ^and 10^4 ^cfu/g faeces [30]. With this definition, the long-term shedder in the study would qualify as a high-shedder. It has previously been observed that calves colonised by direct contact transmission from calf to calf excrete *E. coli *O157:H7 at levels ranging from <30 to 10^6 ^cfu/g for up to 6 weeks [19]. This was supported in the present study, where both of the non-inoculated calves began faecal excretion after a few days. One of these calves shed bacteria in similarly high numbers and for a similarly long period as the inoculated calves.

At a point when no positive samples had been obtained from Stall B for three weeks, Calf V was re-inoculated with an equally high dose of CCUG 53931 as used in the first inoculation. Calf VI in the same stall, which had been naturally infected and had shed intermittently at low levels, was also challenged with the same infectious dose. After challenge, Calf V shed the bacterium for a similar period as after the first inoculation; approximately 11 days. However, after the first inoculation the shedding persisted at levels of approximately 10^5 ^cfu/g for 7 days (even with a small initial upturn) whereas after re-inoculation the numbers shed declined more rapidly and within 3 days reached levels of approximately 10^4 ^cfu/g. Thus, there is some indication that the total number of bacteria shed after re-infection was reduced compared to the first challenge. This is consistent with results obtained by Naylor et al [28] who demonstrated that re-infection of calves with a homologous strain led to a humoral response that could reduce the number of bacteria shed. On the other hand, after the high challenge dose, calf VI exhibited no shedding pattern indicative of any protective immunity. We therefore speculate that the first exposure, which led to the natural infection with intermittent low-level shedding from calf VI, only induced a transient colonisation that was insufficient to produce a protective immunity.

In this study the bacterium was administered orally. It has been suggested that the recto-anal junction mucosa (RAJ) is the site of *E. coli *O157:H7 colonisation in cattle [31] and that rectal administration of a high dose *E. coli *O157:H7 in cattle more effectively produces an experimental colonisation than does oral administration [32]. All calves in this experiment shed the bacterium following oral administration in numbers indicative of proliferation. It is difficult to determine if this is equivalent with colonisation. However, the shedding pattern observed in calf II indicates that this calf was successfully colonised.

Occasionally, a positive faecal sample was obtained from a previously culture-negative animal. This might have been because the calf excreted bacterial numbers below the detection limit of the IMS method, which was calculated to approx. 1 cfu/g faeces under these experimental conditions (unpublished data). It could also have been caused by intermittent excretion or to spontaneous re-infection by direct contact transmission from the other calves or the environment.

There were no findings of VTEC O157:H7 in samples from the pharynx, thus corroborating another study [24] where *E. coli *O157:H7 was not isolated from the oral cavity. In view of the findings in the present study, i.e. that some animals at the same time were faecal culture-positive, the inoculation dose was high and the environment was contaminated with VTEC O157:H7, one would expect to find VTEC O157:H7 in the pharynx. However, if shedding is intermittent, presence in the oral cavity may also be intermittent. In contrast to the present study, Keen and Elder reported that *E. coli *O157 was detected in more than 70% of samples collected from the oral cavity of beef cattle in one feedlot [23], but results from feedlot cattle may not be readily applicable to Swedish dairy calves.

A method for enumerating VTEC O157:H7 was developed by direct plating of dilutions of faeces on ChromO157 plates supplemented with potassium tellurite. VTEC O157:H7 was recovered in satisfactory numbers and the background flora was suppressed so that the VTEC O157:H7 colonies became prominent and showed a distinct morphological pattern with pink colour that could readily be recognized and counted on the agar plate. A very close correlation between VTEC O157:H7 and the formation of pink-coloured colonies on ChromO157 plates has been described earlier [33]. Direct plating on Sorbitol McConkey agar plates supplemented with cefixime and potassium tellurite (CT-SMAC) is a common method for counting *E. coli *O157:H7 in experimental studies [29,34,35]. In the present study however, CT-SMAC was found to restrain the growth of the strain substantially and was rejected for that reason (unpublished data). The method described here has only been evaluated for the particular bacterial strain used in this study, and with calves from one single farm. A general method for counting VTEC O157:H7 in cattle faeces by direct plating on ChromO157 plates, would be interesting to investigate further.

PFGE was performed on selected isolates throughout the experiment, to ascertain that the excreted bacteria emanated from the inoculated strain (CCUG 53931). Twentysix out of 28 of the isolates subjected to analysis exhibited the same pulsotype as the inoculum. One possible explanation for finding slightly differing PFGE profiles in two isolates, from 2 calves on day 5 pi of the re-inoculation, is that the original bacterial strain may have changed during passage through the GI tract of one of the calves. There are reports of clonal turnover of VTEC O157:H7 colonising cattle [36,37]. The finding that both calves in stall B harboured the differing pulsotypes could be due to contact transmission from one calf to the other. Interestingly, when VTEC O157:H7 (PT4;*vtx*_2;_*vtx*_2c_) is isolated from Swedish human cases a predominant pulsotype is found, but many closely related subtypes to this pulsotype have also been demonstrated [8]. Possibly, these different subtypes arise due to clonal turnover within the GI tract of colonised cattle. Likewise, the clonal turnover may also take place in the human GI tract.

## Conclusion

This study contributes with information concerning the dynamics of a specific subtype of VTEC O157:H7 colonisation in dairy calves. This subtype, VTEC O157:H7 (PT4;*vtx*_2;_*vtx*_2c_), is frequently isolated from Swedish cattle and has also been found to cause the majority of reported human infections in Sweden during the past 15 years. In most calves, inoculated with a representative strain of this specific subtype, the numbers of shed bacteria declined over the first two weeks. One calf could possibly be classified as a high-shedder, excreting high levels of the bacterium for a prolonged period.

The overall results agree with other experimental infection studies in cattle. Thus, this VTEC O157:H7 subtype probably does not exhibit any differences in shedding pattern, neither in number of bacteria shed, nor in duration of shedding, compared to other VTEC O157:H7 described in the literature. Further investigations are needed to establish if this particular subtype harbours certain traits that distinguish it from other VTEC O157:H7 strains.

## Competing interests

The authors declare that they have no competing interests.

## Authors' contributions

The study was designed by MEJ, EE and AA. MEJ, EE and AA did the laboratory work and the analysis of results was done by all authors. MEJ drafted the manuscript and all authors revised, read and approved the final manuscript.

## References

[B1] Borczyk AA, Karmali MA, Lior H, Duncan LM (1987). Bovine reservoir for verotoxin-producing *Escherichia coli* O157:H7. Lancet.

[B2] Zhao T, Doyle MP, Shere J, Garber L (1995). Prevalence of enterohemorrhagic *Escherichia coli* O157:H7 in a survey of dairy herds. Appl Environ Microbiol.

[B3] Paiba GA, Gibbens JC, Pascoe SJ, Wilesmith JW, Kidd SA, Byrne C, Ryan JB, Smith RP, McLaren M, Futter RJ (2002). Faecal carriage of verocytotoxin-producing *Escherichia coli* O157 in cattle and sheep at slaughter in Great Britain. Vet Rec.

[B4] Martin ML, Shipman LD, Wells JG, Potter ME, Hedberg K, Wachsmuth IK, Tauxe RV, Davis JP, Arnoldi J, Tilleli J (1986). Isolation of *Escherichia coli* O157:H7 from dairy cattle associated with two cases of haemolytic uraemic syndrome. Lancet.

[B5] Chapman PA, Siddons CA, Wright DJ, Norman P, Fox J, Crick E (1993). Cattle as a possible source of verocytotoxin-producing *Escherichia coli* O157 infections in man. Epidemiol Infect.

[B6] Rice DH, Hancock DD, Vetter RL, Besser TE (1996). *Escherichia coli* O157 infection in a human linked to exposure to infected livestock. Vet Rec.

[B7] Jackson SG, Goodbrand RB, Johnson RP, Odorico VG, Alves D, Rahn K, Wilson JB, Welch MK, Khakhria R (1998). *Escherichia coli* O157:H7 diarrhoea associated with well water and infected cattle on an Ontario farm. Epidemiol Infect.

[B8] Löfdahl  S (2008). How global is VTEC?. Epidemiology and Transmission of VTEC and other Pathogenic Escherichia coli; Stockholm, Sweden.

[B9] Löfdahl  S, Häggman  S, Ramberg M, Jong Bd, Andersson Y (2001). Shiga toxin producing *E. coli *O157:H7 in Sweden seem to have national characteristics. Concerted action CT98-9393 Verocytotoxigenic Ecoli in Europe; Dublin, Ireland.

[B10] Sartz L, De Jong B, Hjertqvist M, Plym-Forshell L, Alsterlund R, Löfdahl  S, Osterman B, Ståhl A, Eriksson E, Hansson HB, Karpman D (2007). An outbreak of *Escherichia coli* O157:H7 infection in southern Sweden associated with consumption of fermented sausage; aspects of sausage production that increase the risk of contamination. Epidemiol Infect.

[B11] Söderström A, Osterberg P, Lindqvist A, Jönsson B, Lindberg A, Blide Ulander S, Welinder-Olsson C, Löfdahl  S, Kaijser B, De Jong B (2008). A large *Escherichia coli* O157 outbreak in Sweden associated with locally produced lettuce. Foodborne Pathog Dis.

[B12] Boqvist S, Aspan A, Eriksson E (2009). Prevalence of verotoxigenic *Escherichia coli* O157:H7 in fecal and ear samples from slaughtered cattle in Sweden. J Food Prot.

[B13] Eriksson E, Aspan A, Gunnarsson A, Vågsholm I (2005). Prevalence of verotoxin-producing *Escherichia coli* (VTEC) 0157 in Swedish dairy herds. Epidemiol Infect.

[B14] Kistemann T, Zimmer S, Vågsholm  I, Andersson Y (2004). GIS-supported investigation of human EHEC and cattle VTEC O157 infections in Sweden: geographical distribution, spatial variation and possible risk factors. Epidemiol Infect.

[B15] Cray WC, Moon HW (1995). Experimental infection of calves and adult cattle with *Escherichia coli* O157:H7. Appl Environ Microbiol.

[B16] Hancock DD, Besser TE, Rice DH, Herriott DE, Tarr PI (1997). A longitudinal study of *Escherichia coli* O157 in fourteen cattle herds. Epidemiol Infect.

[B17] Shaw DJ, Jenkins C, Pearce MC, Cheasty T, Gunn GJ, Dougan G, Smith HR, Woolhouse ME, Frankel G (2004). Shedding patterns of verocytotoxin-producing *Escherichia coli* strains in a cohort of calves and their dams on a Scottish beef farm. Appl Environ Microbiol.

[B18] Ohya T, Ito H (1999). Experimental infection of calves with *Escherichia coli* O157:H7. J Vet Med Sci.

[B19] Besser TE, Richards BL, Rice DH, Hancock DD (2001). *Escherichia coli* O157:H7 infection of calves: infectious dose and direct contact transmission. Epidemiol Infect.

[B20] Brown CA, Harmon BG, Zhao T, Doyle MP (1997). Experimental *Escherichia coli* O157:H7 carriage in calves. Appl Environ Microbiol.

[B21] Sanderson MW, Besser TE, Gay JM, Gay CC, Hancock DD (1999). Fecal *Escherichia coli* O157:H7 shedding patterns of orally inoculated calves. Vet Microbiol.

[B22] Chase-Topping M, Gally D, Low C, Matthews L, Woolhouse M (2008). Super-shedding and the link between human infection and livestock carriage of *Escherichia coli* O157. Nat Rev Microbiol.

[B23] Keen JE, Elder RO (2002). Isolation of shiga-toxigenic *Escherichia coli* O157 from hide surfaces and the oral cavity of finished beef feedlot cattle. J Am Vet Med Assoc.

[B24] Dunn JR, Keen JE, Thompson RA (2004). Prevalence of Shiga-toxigenic *Escherichia coli* O157:H7 in adult dairy cattle. J Am Vet Med Assoc.

[B25] Gautom RK (1997). Rapid pulsed-field gel electrophoresis protocol for typing of *Escherichia coli* O157:H7 and other gram-negative organisms in 1 day. J Clin Microbiol.

[B26] Paton AW, Paton JC (1998). Detection and characterization of Shiga toxigenic *Escherichia coli* by using multiplex PCR assays for stx1, stx2, eaeA, enterohemorrhagic E. coli hlyA, rfbO111, and rfbO157. J Clin Microbiol.

[B27] Gannon VP, D'Souza S, Graham T, King RK, Rahn K, Read S (1997). Use of the flagellar H7 gene as a target in multiplex PCR assays and improved specificity in identification of enterohemorrhagic *Escherichia coli* strains. J Clin Microbiol.

[B28] Naylor SW, Flockhart A, Nart P, Smith DG, Huntley J, Gally DL, Low JC (2007). Shedding of *Escherichia coli* O157:H7 in calves is reduced by prior colonization with the homologous strain. Appl Environ Microbiol.

[B29] Shere JA, Kaspar CW, Bartlett KJ, Linden SE, Norell B, Francey S, Schaefer DM (2002). Shedding of *Escherichia coli* O157:H7 in dairy cattle housed in a confined environment following waterborne inoculation. Appl Environ Microbiol.

[B30] Fox JT, Renter DG, Sanderson MW, Nutsch AL, Shi X, Nagaraja TG (2008). Associations between the presence and magnitude of *Escherichia coli* O157 in feces at harvest and contamination of preintervention beef carcasses. J Food Prot.

[B31] Naylor SW, Roe AJ, Nart P, Spears K, Smith DG, Low JC, Gally DL (2005). *Escherichia coli* O157: H7 forms attaching and effacing lesions at the terminal rectum of cattle and colonization requires the LEE4 operon. Microbiology.

[B32] Sheng H, Davis MA, Knecht HJ, Hovde CJ (2004). Rectal administration of *Escherichia coli* O157:H7: novel model for colonization of ruminants. Appl Environ Microbiol.

[B33] Bettelheim KA (1998). Reliability of CHROMagar O157 for the detection of enterohaemorrhagic *Escherichia coli* (EHEC) O157 but not EHEC belonging to other serogroups. J Appl Microbiol.

[B34] Wray C, McLaren IM, Randall LP, Pearson GR (2000). Natural and experimental infection of normal cattle with *Escherichia coli* O157. Vet Rec.

[B35] Woodward MJ, Gavier-Widen D, McLaren IM, Wray C, Sozmen M, Pearson GR (1999). Infection of gnotobiotic calves with *Escherichia coli *o157:h7 strain A84. Vet Rec.

[B36] Akiba M, Sameshima T, Nakazawa M (2000). Clonal turnover of enterohemorrhagic *Escherichia coli* O157:H7 in experimentally infected cattle. FEMS Microbiol Lett.

[B37] Sanderson MW, Sargeant JM, Shi X, Nagaraja TG, Zurek L, Alam MJ (2006). Longitudinal emergence and distribution of *Escherichia coli* O157 genotypes in a beef feedlot. Appl Environ Microbiol.

